# Supplementary material for: WordCommentsAnalyzer: A windows software tool for qualitative research
Source: F1000Res. 2018 Sep 4;7:536. Originally published 2018 May 3. [Version 2] doi: 10.12688/f1000research.14819.2 (PMC6137411; doi:10.12688/f1000research.14819.2)
Supplement: Supplementary file 3 [file f1000research-7-17614-s0002.tgz › b2a79496-4ada-47c4-9682-a99ce867836d.zip!/Eight Iranian Health Journals/Journal of Health System Research _8.docx]

© Journal of Health System Research . This work is licensed under CC BY-NC 4.0 (https://creativecommons.org/licenses/by-nc/4.0/).

Volume & Issue: دوره 12، شماره 2، 1395

Title: بررسی آمادگی، آگاهی و درک ریسک کارکنان دانشگاه علوم پزشکی شیراز در برابر زلزله در سال 1392

http://hsr.mui.ac.ir/index.php/jhsr/article/view/1988

مقدمه: آگاهی، درک ریسک و آمادگی افراد در برابر زلزله، نقش بسیار حیاتی در کاهش پیامدهای ناشی از آن دارد. این مطالعه با هدف ارزیابی میزان آمادگی، آگاهی و درک ریسک کارکنان دانشگاه علوم پزشکی شیراز در برابر زلزله انجام شد. روش‌ها: این مطالعه مقطعی بر روی 215 نفر از کارکنان دانشگاه علوم پزشکی شیراز صورت گرفت که به طور تصادفی انتخاب گردیدند. ابزار جمع‌آوری اطلاعات، پرسش‌نامه محقق ساخته‌ای شامل اطلاعات دموگرافیک، درک ریسک، آگاهی و آمادگی افراد در برابر زلزله بود. داده‌ها در نرم‌افزار SPSS مورد تجزیه و تحلیل قرار گرفت. سطح معنی‌داری 05/0 < P در نظر گرفته شد. یافته‌ها: بیشتر افراد مورد بررسی زن (54 درصد)، دارای تحصیلات دیپلم و بالاتر (85 درصد)، در گروه سنی 40-30 سال (50 درصد) و متأهل (75 درصد) بودند. درک ریسک و آمادگی اغلب افراد در سطح متوسط و میزان آگاهی اغلب آن‌ها در سطح بالا ارزیابی شد. نتیجه‌گیری: افراد شرکت کننده مطالعه حاضر از سطح تحصیلات بالایی برخوردار بودند و به خاطر رشته تحصیلی و ماهیت شغلی خود، آموزش‌هایی را در زمینه آمادگی در برابر زلزله دریافت نمودند. با این حال، میزان آمادگی آن‌ها در برابر زلزله در سطح متوسط ارزیابی شد. با توجه به این‌ که میزان آمادگی عموم مردم در برابر زلزله در سطوح پایین‌تری قرار دارد، لازم می‌باشد که مطالعات مشابهی در حجم نمونه بالا و در سطح عموم مردم جامعه صورت گیرد تا بر اساس نتایج حاصل از آن، نسبت به ارتقای وضعیت موجود اقدام شود.

Title: برآورد تعداد موارد مرگ و بستری در بیمارستان به علت بیماری‌های قلبی- عروقی و تنفسی منتسب به 10PM در هوای هفت کلان‌شهر ایران

http://hsr.mui.ac.ir/index.php/jhsr/article/view/2207

مقدمه: از دیدگاه مخاطرات بهداشت و سلامت عمومی، ذرات معلق از آلاینده‌های اصلی هوا می‌باشند که با میزان بالایی از مرگ و میر در مواجهه‌های بلند و کوتاه مدت مرتبط هستند. بنابراین، مطالعه حاضر با هدف برآورد اثرات بهداشتی منتسب به 10PM در هفت شهر مشهد، تبریز، اصفهان، شیراز، اراک، اهواز و ارومیه در سال 1390 توسط مدل AirQ انجام شد. روش‌ها: در این مطالعه مقطعی جهت دستیابی به اهداف مطالعه، ابتدا داده‌های آلاینده 10PM از سازمان محیط زیست شهرهای مورد مطالعه اخذ گردید. بعد از اعتبارسنجی داده‌ها، شاخص‌های آماری مورد نیاز جهت کمی‌سازی اثرات بهداشتی محاسبه گردید. داده‌های پردازش شده وارد مدل AirQ شد و نتایج به صورت موارد مرگ و میر برای شهرهای مورد مطالعه ارایه گردید. یافته‌ها: متوسط غلظت سالیانه 10PM در شهرهای اهواز و اصفهان بیشترین مقدار را داشت و به ترتیب 193 و 127 (3µg/m) بود که به طور تقریبی 65/9 و 35/6 برابر استاندارد ایران و سازمان بهداشت جهانی (WHO یا World Health Organization) (3µg/m 20) می‌باشد. تعداد تجمعی موارد کل مرگ منتسب به 10PM به ترتیب در شهرهای مشهد، تبریز، اصفهان، شیراز، اراک، اهواز و ارومیه برابر 634، 302، 542، 362، 123، 549 و 169 نفر برآورد گردید. از کل 122282 مورد بستری در بیمارستان به دلیل بیماری‌های تنفسی در هفت شهر، حدود 8144 مورد به 10PM نسبت داده شد. نتیجه‌گیری: پژوهش حاضر بر تأثیر نامطلوب ذرات معلق بر سلامت انسان تأکید دارد. بنابراین، لزوم برنامه‌ریزي درست و انجام اقدامات مؤثر جهت کاهش اثرات سوء آن بر سلامت عموم و کنترل آثار مخرب آلاینده‌هاي هوا از جمله ذرات معلق را آشکار می‌سازد.

Title: شناسایی ترکیبات حد واسط اکسیداسیون و بررسی تغییرات حد سمیت طی فرایند ازن‌زنی کاتالیزوری غیر همگن با کاتالیست نانوذرات اکسید منیزیم جهت حذف مترونیدازول از محلول آبی

http://hsr.mui.ac.ir/index.php/jhsr/article/view/2240

مقدمه: مترونیدازول (Metronidazole) یکی از پرمصرف‌ترین آنتی‌بیوتیک‌های جهان علاوه بر خواص مختلف، دارای اثرات سوء قابل توجهی بر محیط زیست و جانداران می‌باشد. فرایند ازن‌زنی کاتالیزوری نیز یکی از موفق‌ترین روش‌های حذف آنتی‌بیوتیک‌ها به شمار می‌رود، اما این فرایند نیز مانند سایر فرایندهای اکسیداسیون، محصولات جانبی تولید می‌کند که می‌تواند باعث سمیت پساب گردد. روش‌ها: نمونه‌ای از راکتور بعد از 10 دقیقه ازن‌زنی تحت شرایط بهینه برای فرایند ازن‌زنی کاتالیزوری با کاتالیست نانوذرات اکسید منیزیم (MgO) برداشته شد و مواد حد واسط به وسیله دستگاه GC-MS مورد شناسایی قرار گرفتند. میزان معدنی‌سازی فرایند نیز با انجام آزمایش TOC (Total organic carbon) تعیین گردید و تغییرات سمیت با استفاده از زیست آزمونی با دافنیا مگنا بررسی شد. یافته‌ها: 4 ترکیب به عنوان مواد حد واسط اکسیداسیون مورد شناسایی قرار گرفت و میزان معدنی‌سازی فرایند حدود 5/93 درصد به دست آمد. واحد سمیت 48 ساعته نیز از 24/1 ورودی راکتور به 32/0 پساب فرایند کاهش یافت. نتیجه‌گیری: میزان معدنی‌سازی بالایی در فرایند به دست آمد، اما این معدنی‌سازی کامل نبود و موجب تبدیل محلول ورودی از حالت سمی به اندکی سمی شد. در نتیجه می‌توان گفت که فرایند ازن‌زنی کاتالیزوری با نانوذرات اکسید منیزیم جهت حذف مترونیدازول، از نظر میزان معدنی‌سازی و کاهش سمیت فرایندی قابل قبول می‌باشد.

Title: بررسی سمیت احتمالی فاضلاب سه نوع از صنایع آبکاری فلزات بر باکتری‌های رایج در واحد رآکتور منقطع متوالی ‏(SBR) تصفیه‌خانه فاضلاب های صنعتی

http://hsr.mui.ac.ir/index.php/jhsr/article/view/2325

مقدمه: مواد سمی ورودی به تصفیه‌خانه‌های فاضلاب، باعث اختلال در واحدهای بیولوژیکی می‌گردند. بنابراین، شناخت منابع تولید کننده فاضلاب‌های سمی ضروری است. در اين مطالعه، سميت پساب سه نوع از صنایع آبکاری با استفاده از باکتری‌های واحد رآکتور منقطع متوالی (Sequencing Batch Reactor یا SBR) مورد بررسي قرار گرفت. روش‌ها: جهت تعیین میزان بازدارندگی رشد در هر غلظت، از 5/0 سی‌سی‌ باکتری‌های واحد SBR کشت داده شده در محیط نوترینت براث (Nutrient broth) بر روی محیط کشت نوترینت آگار استفاده شد. داده‌ها توسط آناليز Probit در نرم‌افزار SPSS مورد تجزیه و تحلیل قرار گرفت و توسط آن، EC50 (50% Effective concentration) محاسبه گردید. یافته‌ها: بر اساس یافته‌های حاصل شده از پساب‌های نوع اول، دوم و سوم، به ترتیب غلظت‌های 1، کمتر از 50 و کمتر از 100 میلی‌لیتر بر لیتر تأثیری در رشد باکتری‌های مورد آزمایش نداشت؛ در حالی که غلظت 1000 میلی‌لیتر بر لیتر از هر نوع پساب، باعث مرگ تمام گونه‌های باکتریایی مورد آزمایش گردید. بيشترين و کمترين مقادير EC50 در سه نوع پساب‌ به ترتيب در نمونه‌های 3 (960 میلی‌لیتر بر لیتر) و 1 (5/6 میلی‌لیتر بر لیتر) مشاهده شد. نتیجه‌گیری: نتایج مطالعه حاضر نشان داد که پساب بعضی از صنایع آبکاری حتی در کمترین غلظت‌ها نیز می‌تواند اثرات مضری بر جمعیت باکتریایی واحدهای بیولوژیکی تصفیه‌خانه‌ها داشته باشد. بنابراین، توصیه می‌شود تا با توجه به نوع فرایند این صنایع، استانداردهای تخلیه‌ای و قوانین تصفیه در محل برای آن‌ها وضع گردد.

Title: سقوط یک معضل پنهان بهداشتی؛ بررسی روند و میزان شیوع سقوط و عوامل مرتبط با آن در منازل شهر تبریز طی سال‌های 1386 تا 1390

http://hsr.mui.ac.ir/index.php/jhsr/article/view/2248

مقدمه: تاکنون مطالعه سیستماتیکی به منظور ارزیابی میزان فراوانی، علل و آسیب‌هاي جسمی ناشی از سقوط در منازل شهري و روستایی تبریز صورت نگرفته است. بنابراین، مطالعه حاضر جهت مشخص نمودن وضعیت مشکل بهداشتی مذکور در تبریز طراحی و اجرا گردید. روش‌ها: مطالعه حاضر از نوع گذشته‌نگر بود که به شیوه توصیفی- تحلیلی انجام گرفت و در آن، 43170 نفر که به علت سقوط در طول سال‌های 90-1386 به اورژانس بیمارستان‌ها منتقل شده بودند، مورد بررسی قرار گرفتند. برای جمع‌آوری داده‌ها، از سيستم اطلاعات برنامه کشوری ثبت حوادث در جوامع ایمن استفاده گردید. اطلاعات افراد حادثه دیده شامل سن، جنس، سال، ماه، مكان وقوع و نتيجه حادثه بود. داده‌ها با استفاده از آمار توصیفی و آزمون‌های 2c و Independent t مورد تجزیه و تحلیل قرار گرفت. یافته‌ها: 43170 نفر در طول پنج سال به علت آسیب ناشی از سقوط به اورژانس‌ها مراجعه کرده بودند که از این تعداد، 6/56 درصد سقوط‌ها در منزل رخ داده بود. میزان حادثه در بین زنان (9/52 درصد) شایع‌تر از مردان (1/47 درصد) بود. بيشترین میزان فراوانی حوادث به ترتیب در کودکان زیر 10 سال (8/26 درصد) و افراد جوان 29-20 سال (6/16 درصد) اتفاق افتاده بود. بيشترين میزان حوادث در تمامی گروه‌های سنی به جز افراد بالای 70 سال (بیشترین میزان در زمستان)، در فصل تابستان و کمترین میزان در فصل زمستان مشاهده شد. نتیجه‌گیری: بر طبق نتایج مطالعه، بیش از یک دوم حوادث سقوط در جامعه در منازل رخ می‌دهد. بنابراین، توصیه می‌شود با طراحی برنامه‌های آموزشی مناسب و طراحی محیط منازل با استفاده از اصول ارگونومیک، میزان حوادث سقوط را کاهش داد.

Title: پایش میکرو ارگانیسم‌های شاخص و پاتوژن طی فرایند کمپوست سازی از لجن فاضلاب هضم شده بی‌هوازی

http://hsr.mui.ac.ir/index.php/jhsr/article/view/2365

مقدمه: تغییرات میکرو ارگانیسم‌های شاخص و پاتوژن در فرایند کمپوست سازی، روند این فرایند و کیفیت محصول نهایی را مشخص می‌کند. هدف مطالعه حاضر، تعیین تغییرات جمعیت میکرو ارگانیسم‌های شاخص و پاتوژن طی فرایند کمپوست سازی لجن فاضلاب هضم شده به صورت بی‌هوازی بود. روش‌ها: سه توده به روش ویندرو با اختلاط لجن فاضلاب هضم شده به صورت بی‌هوازی با زایدات سبز باغبانی به عنوان عامل حجیم کننده به نسبت‌های حجمی 1 به 1، 1 به 2 (1/2) و 1 به 3 (1/3) تهیه شد. یک توده لجن فاضلاب به عنوان شاهد بدون هر عامل حجیم کننده‌ای آماده گردید. نمونه‌برداری از توده‌های کمپوست طی سه ماه فرایند کمپوست سازی صورت گرفت. نمونه‌های کمپوست جهت تعیین پارامترهای فیزیکو شیمیایی و میکروبی آزمایش شدند. یافته‌ها: نتایج حاصل از بررسی دما نشان داد که توده لجن به مرحله ترموفیلیک نرسید و توده 1/1 نیز فاز ترموفیلیک کوتاه‌تری را نسبت به توده 1/2 و 1/3 طی کرد. تعداد کلیفرم‌های کل، مدفوعی و استرپتوکوک مدفوعی در ابتدای فرایند کمپوست سازی به ترتیب MPN/gDW (MPN در گرم وزن خشک) 109، 108 و 105 در توده‌های 1/2 و 1/3 بود. تعداد میکرو ارگانیسم‌ها در طول فاز ترموفیلیک کاهش یافت و به حد بسیار پایین یا غیر قابل تشخیص برای کلیفرم‌های مدفوعی و حدود 100 برای استرپتوکوک‌های مدفوعی در توده‌های فوق رسید. میزان سالمونلا در هر دو توده با افزایش دما، کاهش یافت و بعد 19 و 47 روز از آغاز فرایند کمپوست سازی به ترتیب در توده 1/3 و 1/2 به حد غیر قابل تشخیص رسید. نتیجه‌گیری: بررسی تغییرات میکروبی طی فرایند کمپوست سازی و محصول نهایی نشان داد که توده‌های 1/2 و 1/3 در دستیابی به فاز ترموفیلیک و نابودی میکرو ارگانیسم‌های بیماری‌زا موفق بودند. بنابراین، استفاده از این کمپوست در زمین‌های کشاورزی، چمن‌ها و باغ‌ها از نظر میکروبی بلامانع می‌باشد.

Title: بررسی ارتباط عوامل تاثیر گذار با حداکثر اکسیژن مصرفی (VO2max) کارگران معدنی در اصفهان

http://hsr.mui.ac.ir/index.php/jhsr/article/view/2386

مقدمه: اندازه‌گيري بيشترين ظرفيت هوازي (VO2max) در ايجاد تناسب فيزيولوژيك بين كارگر و كار اهميت دارد. بدين ترتيب، علاوه بر حفظ تندرستي و توانايي جسمی، ميزان توليد و بهره‌وری نيز بیشتر خواهد شد. مطالعه حاضر با هدف برآورد ظرفيت هوازي و تعيين عوامل مؤثر بر آن در کارگران معدن انجام شد. از آن‌جایی که برآورد حداکثر ظرفیت هوازی کارگران معدن در ایران برای اولین بار در این مطالعه انجام گردید، ثبت این داده‌ها در بانک اطلاعات داده‌های کشوری بسیار مفید خواهد بود. روش‌ها: در مطالعه مقطعی حاضر، 85 نفر از کارگران یک معدن زیرزمینی در استان اصفهان مورد بررسی قرار گرفتند و اطلاعات دموگرافیک و شاخص‌های قد، وزن، شاخص توده بدنی (Body mass index یا BMI) و ضربان قلب آنان اندازه‌گیری و ثبت گردید. میزان VO2max نیز بر اساس نموگرام Astrand‏ ‏و با استفاده از دوچرخه ارگومتر محاسبه شد. در نهایت، ارتباط متغیرهای ثبت شده با ظرفیت هوازی با استفاده از آزمون‌های Independent t و آنالیز رگرسیون در نرم‌افزار SPSS مورد تجزیه و تحلیل قرار گرفت. یافته‌ها: میانگین ظرفیت هوازی در کارگران 86/0 ± 22/4 لیتر در دقیقه به دست آمد. نتایج حاصل از آنالیز رگرسیون نشان داد که بین ظرفیت هوازی با BMI، سن و سابقه کاری ارتباط معنی‌داری وجود نداشت. همچنین، بر اساس نتایج آزمون t، بین ظرفیت هوازی با سیگار کشیدن (036/0 = P) و ورزش کردن (001/0 > P) رابطه معنی‌داری مشاهده شد. نتیجه‌گیری: نتایج مطالعه نشان داد که BMI شاخص مناسبی برای پیش‌بینی حداکثر اکسیژن مصرفی و به دنبال آن برآورد ظرفیت انجام کار فیزیکی نیست. همچنین، نتایج بیان می‌کند که انجام فعالیت منظم ورزشی و عدم استعمال دخانیات، تأثیر بسزایی بر حداکثر اکسیژن مصرفی کارگران معدن دارد.

Title: بررسی ارتباط شاخص استرین ادراکی با شاخص‌های نمره‌ استرین گرمایی، دمای تر گوی‌سان و دمای هوا در شرایط گرم آزمایشگاهی

http://hsr.mui.ac.ir/index.php/jhsr/article/view/2411

مقدمه: در بسیاری از مشاغل، افراد در معرض استرین حرارتی قرار دارند و ارزیابی میزان مواجهه با گرما امری ضروری است. مطالعه حاضر با هدف اعتبارسنجی و بررسی ارتباط شاخص استرین ادراکی ‏(‏Perceptual strain index‏ یا ‏PeSI‏) ‏با دیگر شاخص‌های گرمایی در شرایط گرم آزمایشگاهی انجام شد. روش‌ها: اين مطالعه از نوع تجربی- آزمایشگاهی بود که به‌ صورت مقطعی در پنج شرایط دمایی مختلف (21، 24، 27، 30 و 35 درجه سانتی‌گراد) و بر روی 15 دانشجوی مرد، در اتاقک تنش حرارتی و بر روی تردمیل در سه سطح فعالیت سبک (4/2 کیلومتر بر ساعت)، متوسط (8/4 کیلومتر بر ساعت) و سنگین (3/6 کیلومتر بر ساعت) انجام گرفت. مقیاس احساس گرمایی (Thermal sensation یا TS)، شدت فعالیت اعمال ‌شده ادراکی (Perceived exertion یا PE) جهت محاسبه PeSI و پرسش‌نامه شاخص نمره‌گذاری استرین گرمایی (Heat strain score index یا HSSI) تکمیل گردید. همچنین، شاخص دمای تر گوی‌سان (‏Wet-bulb globe temperature‏ یا ‏WBGT‏)‏ و دمای هوا ثبت شد. در نهایت، داده‌ها با استفاده از آزمون همبستگی Pearson و آنالیز رگرسیون مورد تجزیه و تحلیل قرار گرفت. یافته‌ها: بر اساس آزمون همبستگی Pearson، بین شاخص‌های PeSI و HSSI همبستگی بالایی وجود داشت (84/0 = r، 001/0 = P). همچنین، بین PeSI با شاخص WBGT و دمای هوا به ترتیب همبستگی خوب (76/0= r) و بالایی (81/0= r) مشاهده شد (001/0 = P). نتیجه‌گیری: نتایج مطالعه حاضر نشان داد که می‌توان از PeSI در ارزیابی استرین گرمایی استفاده نمود؛ چرا ‌که همبستگی قابل قبولی با دیگر شاخص‌های معتبر استرس گرمایی دارد و دارای کاربرد آسان و سریع و هزینه بسیار کمتری نسبت به دیگر شاخص‌های ارزیابی استرس گرمایی می‌باشد.

Title: بررسي كارايي رآكتور ناپيوسته متوالي در حذف نيتروژن و فسفر از فاضلاب سنتتیک در مقياس آزمایشگاهی

http://hsr.mui.ac.ir/index.php/jhsr/article/view/2422

مقدمه: رآکتور ناپیوسته متوالی ‏(‏Sequencing batch reactor‏ یا ‏SBR‏)‏ به دلیل سادگی فرايند، بهره‌برداري انعطاف‌پذير براي حذف مواد مغذي و کاربرد در ابعاد مختلف، جزء پركاربردترين سيستم‌های تصفيه فاضلاب به شمار می‌رود. هدف از انجام مطالعه حاضر، ارزیابی كارايي سيستم رآكتورهاي ناپيوسته متوالي در حذف مواد آلی، نيتروژن و فسفر از فاضلاب سنتتیک در مقياس آزمایشگاهی بود. روش‌ها: یک SBR با ظرفيت مفيد 24 ليتر، در يك چرخه كاري ٢۴ ساعته با توالي فازهاي ۳٠ دقيقه پر كردن، ١٨۰ دقيقه هوازي، ۱٢۰ دقيقه بي‌هوازي، ۹۰۰ دقيقه هوازي، ١٨٠ دقيقه بي‌هوازي، 20 دقیقه ته‌نشینی، 5 دقيقه تخليه و 5 دقيقه سکون مورد بهره‌برداري قرار گرفت. غلظت نيتروژن و فسفر ورودي به ترتيب ۴۰ و 20 میلی‌گرم بر لیتر و بار آلي ورودي معادل 41/0 تا 83/0 کیلوگرم COD (Chemical oxygen demand) بر مترمکعب در روز بود. نمونه‌برداري‌ها در مرحله هوازي اول و پس از ساعت سوم بي‌هوازي، انجام شد. یافته‌ها: در اين سيستم، متوسط بازده حذف COD در پساب خروجي به ترتيب 3/65، 9/92، 2/95، 3/93، 0/96 و 5/96 درصد بود. بيشترين میزان بازدهي حذف مواد آلي و فسفر فسفاته در مرحله ششم به ترتيب حدود ٥/۹۶ و ۰/۳۳ درصد و براي نيتروژن آمونياكي در مرحله اول حدود ۶/۳۴ درصد به دست آمد. با افزايش بار آلي، راندمان حذف مواد آلي، نیتروژن و فسفر افزايش يافت. نتیجه‌گیری: نتایج مطالعه حاضر نشان دهنده كارايي مطلوب SBR در حذف مواد آلي و مغذي (نیتروژن و فسفر) می‌باشد.

Title: بررسی کارایی لجن برگشتی زلال‌ساز به عنوان کمک منعقد کننده پلی‌سولفات فریک در حذف کدورت و کلی‌فرم کل از آب رودخانه بهمن‌شیر آبادان

http://hsr.mui.ac.ir/index.php/jhsr/article/view/2460

مقدمه: در مطالعه حاضر، از لجن برگشتی زلال‌ساز به عنوان کمک منعقد کننده پلی‌سولفات فریک (Poly ferric sulfate یا PFS) به منظور بررسی حذف کدورت و کلی‌فرم کل از آب رودخانه بهمن‌شیر آبادان در سال 1393 استفاده گردید. روش‌ها: در این پژوهش، از دستگاه جار به منظور شبیه‌سازی فرایند انعقاد و لخته سازی در مقیاس آزمایشگاهی استفاده شد. نمونه‌های آب از آبگیر تصفیه‌خانه 11 آبادان برداشت گردید و اثر پارامترهای سرعت اختلاط، pH، دوز منعقد کننده، کدورت اولیه و دوز کمک منعقد کننده بر روی بازده حذف کدورت و کل باکتری‌های کلی‌فرم مورد بررسی قرار گرفت. کل باکتری‌های کلی‌فرم نیز بر اساس روش 9 لوله‌ای و دستورالعمل شماره 9221 استاندارد روش انجام گرفت. یافته‌ها: یافته‌ها نشان داد که بالاترین بازده حذف کدورت و کلی‌فرم در دوز 15 میلی‌گرم در لیتر از PFS، سرعت اختلاط 120 دور در دقیقه، 7 = pH و دوز 2 میلی‌لیتر از کمک منعقد کننده به دست آمد. بیشترین بازده حذف هم‌زمان کدورت وکلی‌فرم نیز در دوز 2 میلی‌لیتر از لجن برگشتی حاصل شد و به عنوان دوز بهینه لجن برگشتی انتخاب گردید. حداکثر بازده حذف برابر 97 درصد در کدورت اولیه و برابر با 150 واحد کدورت نفلومتری (Nephelometric Turbidity Units یا NTU) به دست آمد. نتیجه‌گیری: راندمان PFS در کدورت‌های بالاتر افزايش یافت. دوز 2 میلی‌لیتر از لجن برگشتی در غلظت اولیه 15 میلی‌گرم در لیتر از PFS، به عنوان دوز بهینه انتخاب شد؛ چرا که استانداردهای لازم (کدورت کمتر از NTU 5) جهت آب آشامیدنی را تأمین می‌کند.

Title: بررسی اثر ضخامت، شدت صوت و نحوه قرارگیری لایه هوا بر ضریب جذب صوت فوم پلی‌اورتان با استفاده از روش تابع انتقال

http://hsr.mui.ac.ir/index.php/jhsr/article/view/2501

مقدمه: فوم پلی‌اورتان یکی از مواد جاذب صوت می‌باشد که در ایران به عوامل تأثیرگذار بر ضریب جذب صوت آن توجه چندانی نشده است. بنابراین، مطالعه حاضر با هدف بررسی تأثیر ضخامت، وجود لایه هوا و میزان شدت صوت بر میزان جذب صوتی فوم پلی‌اورتان انجام شد. روش‌ها: در این مطالعه، لوله امپدانس طبق استاندارد ساخته شد و سپس ضریب جذب فوم پلی‌اورتان سلول باز (Open cell) با دو ضخامت 25 و 50 میلی‌متر اندازه‌گیری گردید. سپس هر دو ضخامت در 6 شدت صوت مختلف بررسی شد. همچنین، ضریب جذب فوم 25 میلی‌متری با 5/5 سانتی‌متر لایه هوا و فوم 50 میلی‌متری با 3 سانتی‌متر هوا در انتهای نمونه مورد سنجش قرار گرفت. علاوه بر این، اندازه‌گیری جذب برای فوم 40 میلی‌متری یک‌بار به صورت 2 سانتی‌متر فوم، 1 سانتی‌متر هوا و 2 سانتی‌متر فوم و در حالت بعد به صورت 4 سانتی‌متر فوم و 1 سانتی‌متر هوا در انتها انجام گردید. یافته‌ها: یافته‌های تعیین دقت و کارایی مطالعه نشان داد که لوله امپدانس با ضریب همبستگی 98/0 قابل استفاده می‌باشد. شدت صوت در هر دو ضخامت تأثیر چشمگیری در میزان جذب صوت نداشت. همچنین، لایه هوا در انتهای فوم 25 و 50 میلی‌متری سبب افزایش جذب در فرکانس‌های میانی و بالا شد. علاوه بر این، در مقایسه با فوم 5 سانتی‌متر بدون هوا، وجود 1 سانتی‌متر هوا بین فوم 40 میلی‌متری، باعث افزایش جذب در همه فرکانس‌های مورد مطالعه و در حالت 1 سانتی‌متر هوا در انتها سبب افزایش جذب در فرکانس‌های بالا گردید. نتیجه‌گیری: با توجه به نتایج تحقیق، می‌توان ضریب جذب صوت فوم پلی‌اورتان سلول باز را با در نظر گرفتن ضخامت بیشتر با توجه به شرایط موجود و قرار دادن لایه هوا در پشت یا بین ماده جاذب بهبود بخشید.

Title: بررسی قدرت پیشگویی کنندگی نظریه رفتار برنامه‌ریزی شده بر قصد زوجین نابارور در حل مشکل ناباروری

http://hsr.mui.ac.ir/index.php/jhsr/article/view/2638

مقدمه: ناباروری یکی از مشکلات عمده پزشکی در دنیای امروز به شمار می‌رود که میزان آن در سراسر دنیا در حال افزایش است. ناباروری بر مردان و زنان سنین باروری تأثیر می‌گذارد. همچنین، زوجین را در معرض مشکلات عاطفی و روانی فراوانی قرار می‌دهد. نظریه رفتار برنامه‌ریزی شده یکی از مدل‌هایی است که فرایند اصلی پذیرش رفتارهای مناسب را توضیح می‌دهد. مطالعه حاضر با هدف بررسی قدرت پیشگویی کنندگی نظریه رفتار برنامه‌ریزی شده بر قصد زوجین نابارور در حل مشکل ناباروری طراحی شد. روش‌ها: در این مطالعه توصیفی- تحلیلی، 64 نفر از زوجین نابارور مراجعه کننده به مطب متخصصان زنان و نازایی شهر بیرجند، در سال 1394 مورد بررسی قرار گرفتند. افراد به روش نمونه‌گیری در دسترس انتخاب شدند. ابزار جمع‌آوری داده‌ها، پرسش‌نامه محقق ساخته منطبق با سازه‌های نظریه رفتار برنامه‌ریزی شده بود. روایی پرسش‌نامه با استفاده از نظرات 10 نفر از متخصصان و پایایی آن نیز به وسیله ضریب Cronbach¢s alpha تأیید گرديد. برای تعیین چگونگی ارتباط سازه‌ها، از ضریب همبستگی Pearson و جهت تعیین سهم هر یک از سازه‌ها، از روش آنالیز رگرسیون استفاده شد. داده‌ها در نرم‌افزار SPSS مورد تجزیه و تحلیل قرار گرفت. 050/0 > P به عنوان سطح معنی‌داری در نظر گرفته شد. یافته‌ها: میانگین سنی زوجین شرکت کننده 24/5 ± 68/30 سال بود. همبستگی مثبت و معنی‌داری بین تمام متغیرهای مدل رفتار برنامه‌ریزی شده با قصد مشاهده گردید (050/0 > P). نتایج آنالیز رگرسیون مشخص نمود که سازه‌های نظریه رفتار برنامه‌ریزی شده، 36 درصد واریانس قصد را پیشگویی می‌کند. سازه‌های کنترل رفتار درک شده (465/0 = b) و نگرش (289/0 = b)، به ترتیب بهترین پیشگویی کننده قصد حل مشکل ناباروری بودند (050/0 > P).

Title: بررسی توزیع فراوانی میزان باقی‌مانده آنتی‌بیوتیک تتراسایکلین در دو بخش سفیده و زرده تخم‌مرغ‌های عرضه شده در شهر اصفهان

http://hsr.mui.ac.ir/index.php/jhsr/article/view/2606

مقدمه: در سال‌های اخیر، شواهد متعددی از مصرف گسترده آنتی‌بیوتیک‌های گروه تتراسایکلین برای پرورش طیور در برخی از مناطق جهان مشاهده شده است و در برخی موارد، باقی‌مانده این آنتی‌بیوتیک‌ها از حدود مجاز تعیین شده فراتر رفته‌، اما تاکنون گزارش این مورد در ایران منتشر نشده است. روش‌ها: مطالعه حاضر به صورت مقطعی در پاییز سال 1393، بر روی 40 نمونه تخم‌مرغ صنعتی از برندهای مختلف و 6 نمونه تخم‌مرغ بومی که به صورت تصادفی از سطح شهر اصفهان جمع‌آوری شده بود، انجام گرفت. نمونه‌ها از لحاظ باقی‌مانده تتراسایکلین با استفاده از روش کیت اختصاصی ELISA (Enzyme-linked immunosorbent assay) مورد بررسی قرار گرفت. در نهایت، داده‌ها با استفاده از آزمون‌های آماری آنالیز واریانس یک‌طرفه تجزیه و تحلیل گردید. یافته‌ها: محدوده میزان باقی‌مانده تتراسایکلین در نمونه‌های تخم‌مرغ بین 56/0تا ppb 7/9 (میانگین ppb 1/3) به دست آمد. این مقدار در مقایسه با حداکثر مجاز باقی‌مانده (Maximum residue levels یا MRLs) تتراسایکلین در تخم‌مرغ (ppb 200) که توسط کمیته اروپا تعیین شده است، نشان دهنده مقدار مجاز باقی‌مانده آنتی‌بیوتیک در نمونه‌های تخم‌مرغ بود. همچنین، بررسی جداگانه زرده و سفیده نمونه‌ها، بیانگر بالاتر بودن باقی‌مانده دارو در زرده نسبت به سفیده بود. نتیجه‌گیری: نتیجه کلی مطالعه حاکی از ایمن بودن تخم‌مرغ‌های عرضه شده در سطح شهر اصفهان از لحاظ باقی‌مانده آنتی‌بیوتیک تتراسایکلین می‌باشد.

Title: بررسی آلودگی رواناب سطحی حاصل از بارندگی در نقاط پرترافیک شهر همدان به عناصر آهن، سرب و کادمیوم در سال 1392

http://hsr.mui.ac.ir/index.php/jhsr/article/view/2526

مقدمه: رواناب سطحی، جاده‌ای به عنوان منبع خطی انتشار آلودگی با خصوصیات آلایندگی بسیار ویژه و قابلیت تخلیه در محیط زیست محسوب می‌شود. آلودگی فلزات سنگین در رواناب سطحی نیز باعث ایجاد نگرانی‌های فراوانی در قرن گذشته شده است. بنابراین، پژوهش حاضر با هدف بررسی غلظت عناصر آهن، سرب و کادمیوم در رواناب سطحی شهر همدان در سال 1392 انجام گردید. روش‌ها: در این مطالعه از 8 ایستگاه منتخب سطح شهر همدان نمونه‌برداری صورت گرفت و غلظت عناصر در نمونه‌ها بعد از طی مراحل آماده‌سازی آزمایشگاهی، توسط دستگاه ICP-OES ‏(‏Inductively-coupled plasma-atomic emission spectroscopy اندازه‌گیری شد. داده‌ها در نرم‌افزار SPSS مورد تجزیه و تحلیل قرار گرفت. یافته‌ها: میانگین غلظت عناصر آهن، سرب و کادمیوم نمونه‌ها به ترتیب 38/0 ± 42/0، 00/3 ± 23/4 و 46/4 ± 48/6 میکروگرم در لیتر به دست آمد. نتایج آزمون t به منظور مقایسه میانگین غلظت عناصر مورد ارزیابی در نمونه‌ها با رهنمود WHO‏ (‏World Health Organization‏)‏، تفاوت آماری معنی‌داری را نشان داد (05/0 > P)؛ به طوری که میانگین غلظت تمام عناصر بیشتر از حد استاندارد بود. نتیجه‌گیری: نتایج بیانگر آلوده بودن رواناب سطحی مناطق پرترافیک شهر همدان به فلزات آهن، سرب و کادمیوم می‌باشد که منجر به تهدید منابع پذیرنده و به دنبال آن، بروز مخاطرات بهداشتی برای شهروندان می‌شود.

Title: بررسی میزان انتشار ترکیبات BTEX از جایگاه‌های سوخت‌رسانی منطقه خوراسگان اصفهان در سال‌های 93-1392

http://hsr.mui.ac.ir/index.php/jhsr/article/view/2584

مقدمه: بنزن، تولوئن، اتیل بنزن و ایزومرهای گزیلن (Benzene, Toluene, Ethylbenzene, Xylene isomers یا BTEX) از جمله ترکیبات آلی فراری هستند که در رده مواد مشکوک به سرطان‌زایی قرار دارند. این ترکیبات در اثر تبخیر بنزین از جایگاه‌های سوخت‌رسانی، وارد هوا می‌شوند و سلامتی کارکنان، رانندگان و ساکنان اطراف را تحت تأثیر قرار می‌دهند. هدف از انجام این مطالعه، بررسی میزان غلظت BTEX در هوای محیطی جایگاه‌های سوخت‌رسانی و اطراف آن و مقایسه با استانداردهای هوای تنفسی و شغلی بود. روش‌ها: نمونه‌های تحقیق حاضر از هوای کنار پمپ‌های سوخت و 40 متری اطراف آن‌ها به وسیله پمپ نمونه‌برداری فردی و با استفاده از تیوپ‌های کربن فعال جمع‌آوری شد. سپس BTEX توسط حلال دی‌سولفید کربن استخراج گردید و به وسیله دستگاه GC/FID (Gas chromatography-flame ionization detector) مورد سنجش قرار گرفت. جهت نمونه‌برداری و تحلیل داده‌ها، از روش آنالیز هیدروکربن‌های عطری NIOSH (National Institute for Occupational Safety and Health) (روش 1501) استفاده شد. یافته‌ها: میانگین غلظت‌های بنزن، تولوئن، اتیل بنزن و ایزومرهای زایلن در جایگاه‌ها به ترتیب در فصل پاییز 97/235، 22/415، 34/93 و 14/333 میکروگرم در مترمکعب، در فصل زمستان 11/200، 23/329، 34/73 و 48/321 میکروگرم در مترمکعب و در فصل بهار 20/254، 55/432، 26/113 و 17/486 میکروگرم در مترمکعب به دست آمد. غلظت بنزن در جایگاه‌ها بسیار بالاتر از حدود پیشنهاد شده برای تماس شغلی بود. نتیجه‌گیری: بر اساس نتایج به دست آمده، میانگین غلظت BTEX در فصل گرم بیشتر از فصل سرد و میزان انتشار از جایگاه‌های گاز به مراتب کمتر از جایگاه‌های بنزین و گازوئیل می‌باشد. غلظت‌های سنجش شده در فواصل 40 متری از جایگاه‌ها در مقایسه با غلظت‌های خود جایگاه‌ها نیز بسیار پایین بود. بنابراین، انجام اقدامات لازم برای کنترل نشت سوخت از تانک‌ها و پمپ‌ها و امکان بازیافت بخارات بنزین در حین انتقال سوخت، در جهت حفظ سلامتی شاغلان ضروری به نظر می‌رسد.

Title: بررسی فضایی شاخص نیاز به مراقبت‌های سلامت برای سرطان پوست با استفاده از مدل‌سازی معادلات ساختاری در ایران

http://hsr.mui.ac.ir/index.php/jhsr/article/view/2630

مقدمه: محدودیت منابع خدمات سلامت، ضرورت اولویت‌بندی این منابع را ایجاب می‌کند. شاخص نیاز به مراقبت‌های سلامت، جهت بررسی توزیع منابع و خدمات سلامت به کار می‌رود. مطالعه حاضر به برآورد مقادیر این شاخص برای بیماری سرطان پوست با در نظر گرفتن اثر مکان‌های مجاور پرداخت. روش‌ها: این پژوهش از نوع تحقیقات همبستگی بود. جامعه آن از تمامی مبتلایان و فوت شدگان ناشی از بیماری سرطان پوست تشکیل گردید که طی سال‌های 1386 تا 1388 توسط وزارت بهداشت، درمان و آموزش پزشکی به ثبت رسیدند. تجزیه و تحلیل داده‌ها با تکنیک مدل‌سازی معادلات ساختاری و در نرم‌افزار winBUGS انجام شد و ترسیم نقشه‌ جغرافیایی در نرم‌افزار Arc GIS صورت گرفت. یافته‌ها: یافته‌های این پژوهش حاکی از این بود که استان‌های مرکزی، اصفهان، کهگیلویه و بویراحمد به ترتیب بیشترین مقادیر شاخص نیاز به مراقبت‌های سلامت را در مورد بیماری سرطان پوست داشتند. نتیجه‌گیری: نتایج به دست آمده نشان داد که استان‌های واقع در نواحی مرکزی ایران که از ساعات آفتابی بیشتری برخوردارند، نیاز بیشتری به مراقبت‌های سلامت داشتند. بنابراین، با توجه به این‌که مواجهه با تابش آفتاب یک عامل خطر در سرطان پوست می‌باشد، لازم است چنین مکان‌هایی مورد توجه ویژه‌ سیاست‌گذاران عرصه‌ سلامت قرار گیرد. واژه‌های کلیدی: شاخص نیاز به مراقبت‌های سلامت، مدل‌سازی معادلات ساختاری، همبستگی فضایی، سرطان پوست

Title: روند بروز سرطان پوست در استان اصفهان طی سال‌های ۸۷-۱۳۸۳

http://hsr.mui.ac.ir/index.php/jhsr/article/view/2530

مقدمه: شایع‌ترین بدخیمی در دنیا و از جمله در ایران، سرطان پوست می‌باشد. هدف از انجام این مطالعه، تعیین روند بروز سرطان پوست در استان اصفهان بود.  روش‌ها: در مطالعه مقطعی حاضر، از داده‌های سیستم ثبت کشوری موارد سرطان و مرکز مدیریت بیماری‌های وزارت بهداشت، درمان و آموزش پزشکی طی سال‌های ۸۷-۱۳۸۳ استفاده گردید. روند بروز سرطان پوست در هر دو جنس با استفاده از آزمون روند Mantel-Haenszel مورد بررسی قرار گرفت.  یافته‌ها: بروز تطبیق سنی سرطان پوست در سال ۱۳۸۳ در مردان و زنان به ترتیب 4/28 و 3/18 در هر صد هزار نفر و در سال ۱۳۸۷ به ترتیب 8/30 و 9/18 در هر صد هزار نفر بود. در طول سال‌های مطالعه، گروه سنی ۸۴-۸۰ سال بالاترین بروز اختصاصی را در هر دو جنس داشت. ابتلای نسبی سرطان پوست در هر دو جنس در سال ۱۳۸۷نسبت به سال ۱۳۸۳، در مردان و زنان به ترتیب به 08/1 و 03/1 رسید. روند بروز بیماری طی سال‌های مورد مطالعه در مردان (065/0 = P) و زنان (490/0 = P) معنی‌دار نبود. نتیجه‌گیری: با وجود این که روند بروز سرطان پوست در استان اصفهان معنی‌دار نبود، اما افزایش روند بروز و همچنین، بروز دو برابری این سرطان در مردان نسبت به زنان، لزوم آموزش به افراد در زمینه استفاده از وسایل حفاظتی در برابر اشعه فرابنفش خورشید و انجام پژوهش‌های بیشتر در زمینه عوامل خطر بیماری را ضروری می‌سازد. واژه‌های کلیدی: روند بروز؛ سرطان پوست؛ بروز تطبیق سنی

Title: میزان بهره‌مندی از خدمات سلامت توسط جمعیت کارگران ساختمانی و خانواده آن‌ها در شهر اصفهان در سال 1392

http://hsr.mui.ac.ir/index.php/jhsr/article/view/2282

مقدمه: یکی از معیارهای عدالت در سلامت، بهره‌مندی از خدمات سلامت می‌باشد. این مطالعه با هدف تعیین میزان بهره‌مندی از خدمات سلامت در بین کارگران ساختمانی و خانواده آن‌ها به عنوان یکی از آسیب‌پذیرترین اقشار جامعه در مقابل بیماری‌ها و حوادث، انجام شد. روش‌ها: در تحقیق توصیفی- تحلیلی حاضر، میزان بهره‌مندی کارگران ساختمانی و خانواده آنان از خدمات سلامت و عوامل تأثیرگذار بر آن در شهر اصفهان در سال 1392، با استفاده از یک پرسش‌نامه استاندارد مورد ارزیابی قرار گرفت. بدین منظور، برای 400 کارگر ساختمانی متأهل که منتظر به کارگیری روزانه در خدمات ساختمانی بودند و در پیاده‌روهای میدان‌های اصلی چهار گوشه شهر اصفهان مستقر بودند، پرسش‌نامه‌ به صورت سهمیه‌ای، به روش تصادفی ساده و با انجام مصاحبه حضوری تکمیل گردید. یافته‌ها: 5/39 درصد از کارگران ساختمانی تحت پوشش هیچ بیمه درمانی نبودند. 25/19 درصد آنان طی دو هفته اخیر نیاز به خدمات سلامت داشتند که از این خانواده‌ها، تنها 43/71 درصد از این خدمات بهره‌مند شدند. حدود 25/17 درصد از افراد تحت پوشش خانواده کارگران، نیاز به بستری شدن در بیمارستان طی یک سال گذشته را داشتند. متوسط تعداد دفعات بستری در طول سال بین خانواده کارگران، 54/1 بار در سال بود. 75/4 درصد خانوارهای کارگران به دلیل بستری شدن در بیمارستان، متحمل هزینه‌های کمرشکن سلامت شده بودند. نتیجه‌گیری: میزان بهره‌مندی از پوشش بیمه‌های درمانی در بین کارگران، کمتر از میانگین همین نسبت در جامعه ایرانیان می‌باشد. خانواده‌هایی که به علت بستری شدن در بیمارستان متحمل هزینه‌‌های کمرشکن شدند، 5/2 برابر همین نسبت در بین افراد جامعه می‌باشد.

Title: دسترسي جغرافيايي روستاها به خدمات خانه‌های بهداشت روستايي با استفاده از سيستم اطلاعات جغرافيايي (GIS): مطالعه موردي، استان خوزستان

http://hsr.mui.ac.ir/index.php/jhsr/article/view/2039

مقدمه: يكي از چالش‌های اساسي نظام سلامت در بسياري از كشورها، تأمین دسترسي عادلانه و مقرون ‌به ‌صرفه جوامع روستايي به خدمات سلامت است. در اين راستا، سيستم اطلاعات جغرافيايي (Geographical information system یا GIS) ابزار مناسبي براي تحليل و مدل‌سازي دسترسي جغرافيايي به خدمات سلامت در مناطق روستايي به شمار می‌رود. هدف از انجام اين مطالعه، تحليل فضايي دسترسی جغرافیایی روستاهاي استان خوزستان به خانه‌هاي بهداشت روستايي بود. روش‌ها: پژوهش حاضر، كاربردي و از نوع توصيفي- تحليلي بود و جامعه آماری آن را کلیه روستاهاي استان خوزستان در سال 1390 تشكيل داد. براي تحليل فضايي دسترسي روستاهاي استان خوزستان، پس از جمع‌آوري داده‌هاي فضايي و شاخص‌هاي توصيفي و اصلاح آن‌ها، اين داده‌ها وارد نرم‌افزار ArcGIS شد. سپس لايه‌هاي مهم در نرم‌افزار تعريف و محدوده عملياتي خانه‌های بهداشت مشخص گرديد. یافته‌ها: یافته‌های پژوهش نشان داد كه از مجموع 4547 روستاي موجود، 3315 روستا (9/72 درصد) داراي سكنه دایمی، 146 روستا (2/3 درصد) داراي سكنه موسمي (فصلي) و 985 روستا (9/23 درصد) خالي از سكنه بود. فقط 2223 روستا (9/48 درصد) به راه آسفالته دسترسي داشتند. 358 روستاي داراي سكنه دایمي نيز در خارج از حريم خدماتي خانه‌هاي بهداشت روستايي قرار گرفته بودند. نتیجه‌گیری: ‌چالش‌هايي در زمینه دسترسي برخي از روستاهاي استان خوزستان به خانه‌های بهداشت روستايي وجود دارد. برخي از اين چالش‌ها به دلایلی از جمله راه‌های روستايي، دورافتادگی، پراكندگي جغرافيايي روستاها و توسعه نامتعادل روستاها بود. استفاده از GIS مي‌تواند به كارايي بهتر طرح گسترش شبكه كمك كند.

Title: مقایسه دشواری در نظم‌بخشی هیجانی و صفات شخصیت مرزی در معتادان تحت درمان با متادون، معتادان خودمعرف (بدون درمان) و افراد بهنجار

http://hsr.mui.ac.ir/index.php/jhsr/article/view/1866

مقدمه: هدف از انجام پژوهش حاضر، مقایسه دشواری در نظم‌بخشی هیجانی و صفات شخصیت مرزی در معتادان تحت درمان با متادون، معتادان در حال مصرف مواد (خودمعرف) و افراد بهنجار بود. روش‌ها: این پژوهش از نوع مطالعات علی مقایسه‌ای و جامعه آماری آن شامل کلیه مردان سوء مصرف کننده مواد مراجعه کننده به مراکز ترک اعتیاد شهرستان خوی در سال 1392 بود. نمونه آماری پژوهش از 40 نفر معتاد تحت درمان با متادون، 40 نفر معتاد در حال مصرف مواد (خودمعرف) و 40 نفر افراد غیر معتاد تشکیل شد که به صورت در دسترس انتخاب شدند. جهت جمع‌آوری داده‌ها، مقیاس‌های دشواری در نظم‌بخشی هیجانی (Difficulties in Emotion Regulation Scale یا DERS) و صفات شخصیت مرزی (Schizotypal Trait questionnaire-B form یا STB) توسط شرکت کنندگان تکمیل گردید. داده‌ها با استفاده از آزمون‌ تحلیل واریانس چند متغیره (MANOVA) و آزمون تعقیبی Tukey در نرم‌افزار SPSS مورد تجزیه و تحلیل قرار گرفت. یافته‌ها: یافته‌ها نشان داد که افراد خودمعرف (بدون درمان)، نمرات بالاتری در متغیر صفات شخصیت مرزی (05/15 = F، 001/0 = P) و دشواری در نظم‌بخشی هیجانی (47/33 = F، 001/0 = P) نسبت به افراد تحت درمان با متادون و افراد بهنجار کسب کردند، اما نتایج آزمون تعقیبی Tukey نشان داد که تفاوت معنی‌داری بین افراد تحت درمان با متادون و افراد بهنجار در این مؤلفه‌ها وجود نداشت.نتیجه‌گیری: با توجه به نقش دشواری در نظم‌بخشی هیجانی و صفات شخصیت مرزی در اختلالات سوء مصرف مواد، لازم است که پژوهشگران در مطالعات آینده بیشتر بر نقش این متغیرها در شروع و تداوم سوء مصرف مواد توجه داشته باشند. در این راستا، برگزاری کارگاه‌های مربوط به تنظیم هیجانی و مهارت‌های کنترل تکانه برای بیماران سوء مصرف کننده ضروری به نظر می‌رسد
